# Extrachromosomal Circular DNA from TCGA Tumors Is Generated from Common Genomic Loci, Is Characterized by Self-Homology and DNA Motifs near Circle Breakpoints

**DOI:** 10.3390/cancers14092310

**Published:** 2022-05-06

**Authors:** Philip D. Tatman, Joshua C. Black

**Affiliations:** Department of Pharmacology, University of Colorado, Anschutz Medical Campus, Aurora, CO 80045, USA; philip.tatman@cuanschutz.edu

**Keywords:** eccDNA, ecDNA, TCGA, homologous recombination, microhomology mediated recombination, circular DNA, extrachromosomal DNA, extrachromosomal circular DNA, circular DNA

## Abstract

**Simple Summary:**

Extrachromosomal circular DNA is ubiquitous in eukaryotic cells. In tumors, highly amplified oncogenes exist in circular DNA, and circular DNA correlates with poor prognosis in multiple tumor types. Despite the emerging importance of extrachromosomal circular DNA, little is known about the origin or biological function of circular DNA. We investigated publicly available circular DNA from 355 TCGA tumors from 22 tumor types. We identify several locations frequently circularized irrespective of the type of cancer. Analysis of the genes present on circles revealed they are expressed, and at a higher level. These genes were enriched in cancer related functions regardless of tumor type. Analysis of circle breakpoints identified strong presence of homology and microhomology with an enrichment of specific DNA binding transcription factor motifs. Our results provide a framework for addressing key questions in the biogenesis and functional importance of extrachromosomal circular DNA.

**Abstract:**

Extrachromosomal circular DNA has emerged as a frequent genomic alteration in tumors. High numbers of circular DNAs correspond to poor prognosis suggesting an important function in tumor biology. However, despite mounting evidence supporting the importance of circular DNA, little is known about their production, maintenance, or selection. To provide insight into these processes, we analyzed circular DNA elements computationally identified in 355 TCGA tumors spanning 22 tumor types. Circular DNAs originated from common genomic loci irrespective of cancer type. Genes found in circularized genomic regions were more likely to be expressed and were enriched in cancer-related pathways. Finally, in support of a model for circle generation through either a homology or microhomology-mediated process, circles exhibit homology near their breakpoint. These breakpoints are also enriched in specific DNA motifs. Our analysis supports a model where gene-containing circles emerge from common, highly transcribed regions through a homology-mediated process.

## 1. Introduction

Electron micrographs revealed the presence of extrachromosomal circular DNA (eccDNA) in wheat and boar sperm in 1965 [1] and later identified these molecules in HeLa cells [2], demonstrating the existence of eccDNA across the evolutionary spectrum. EccDNA are commonly identified in numerous eukaryotic organisms and human disease [3,4,5,6]. These circular, double-stranded DNAs can originate throughout the genome, ranging in size from tens of basepairs to multiple megabases [3,4,5,6]. However, the biogenesis of these circular DNAs remains unclear. Several non-exclusive mechanisms of origin for eccDNA have been proposed including homologous recombination (HR) [3,7,8,9], the ligation of fragmented apoptotic DNA [10], replication slippage [11], byproducts of DNA double strand break (DSB) repair [3,5,7,8,9], and episome-polymerization [12,13]. Despite many observations confirming the presence of circular DNA in eukaryotes, fundamental questions regarding the mechanism of circular DNA formation, and its biological purpose, remain open.

In humans, circular DNA is best understood in the context of cancer, where it has emerged as an additional mechanism of oncogene amplification [14,15,16]. Circular DNA molecules can harbor oncogenes [16,17,18,19], regulatory elements of oncogenes [17,18], and mutated oncogenes that confer drug resistance [20]. The inclusion and enrichment of both regulatory elements and their corresponding target gene suggest eccDNA may be transcribed or regulate transcription [17]. Moreover, in non-cancerous human tissue, transcripts with specific eccDNA breakpoints were identified [21], further supporting the notion that eccDNA are actively transcribed. This suggests that circular DNA are likely contributing to biological processes and cancer biology, thus understanding their origins may provide insight into cancer development, progression, and expose new therapeutic targets. 

The oncogene-centric evaluation of eccDNA leaves many questions unanswered regarding other genes found in circular DNA. The broad coverage of circular DNA across cancer genomes suggests many genes exist in eccDNA [14,16,22]. However, it is unclear what these genes are, how they contribute to cellular processes in cancer, and whether these genes are commonly found in circular DNA regardless of cancer type or are tumor specific. 

In this study we analyze previously identified circular DNA from 355 TCGA patients from 22 tumor types [22]. Our analysis revealed circular DNAs are produced from common genomic loci regardless of cancer type. Genes on circles are more likely to be expressed and expressed to a higher level than genes outside of circles. Genes commonly found in circles, irrespective of cancer type, exhibit enrichment for metabolic and cancer-related pathways. Consistent with the proposed importance of homology in circular DNA biogenesis, we find evidence of both long stretches of homology and microhomology between circle ends, suggesting HR and microhomology mediated repair (MHMR) mechanisms are important to circle formation in cancer. Finally, we identify enrichment of specific transcription factor motifs at circle ends suggesting sequence-specific DNA binding factors may play a role in the formation, maintenance, or selection of circular DNA. 

## 2. Materials and Methods

### 2.1. Data Acquisition

Previously published circular DNA calls for 355 TCGA tumors from 22 different cancer types were downloaded from the University of Virginia [22]: http://genome.bioch.virginia.edu/TCGA-ATACSEQ-ECCDNA/ (accessed on 1 August 2021). All clinical and molecular TCGA data were acquired through the Xena UCSC genome browser [23]: http://xena.ucsc.edu/ (accessed on 17 December 2021). Relevant code used in this study is provided on GitHub: https://github.com/Black-Lab-UCDenver/CircleAnalysis (made available on 11 April 2021).

### 2.2. Generating Random Circular DNA for Control Comparisons

To generate a representative random sample of circular DNA based on the size distribution and number of DNA circles for each tumor, random BED coordinates were created using the R statistical suite (version 4.1.0). To accomplish this, a random genomic position was selected for each real circle in each tumor sample. Since we removed circles from the TCGA data that included or crossed a centromere, the randomly generated circles were also not allowed to include or cross a centromere [8,24,25]. Genomic positions were iteratively selected until a position was identified that did not cross a centromere or overhang a chromosomal end. If a position selection resulted in coordinates that exceeded chromosome boundaries, or overlapped with a centromere, the process was repeated until all conditions were satisfied. This resulted in 355 random circle sets, one for each tumor sample in the TCGA data set.

### 2.3. Pan-Cancer Genome-Wide Analysis of Circle Location

Genome-wide coverage files of circles and randomly generated circles were calculated for each tumor using BEDtools (version 2.3) [26], and binned to non-overlapping 1 kb bins. To remove the bias of different numbers of circles in each tumor, we transformed the coverage files into a binary format, where “1” indicated the presence of a circle and a “0” indicated no circle. This format allowed us to calculate the total number of tumors (a scale of 0 to 355) with circles or random control circles at each 1 kb bin in the genome. We then determined if the distribution of circles was distinct when compared to random control circles using a Kolmogorov–Smirnov test with a *p*-value less than 0.05. A quantile-quantile (QQ) plot was used to display the differences in the distributions, and kurtosis metrics were used to show the differences in tail volumes. Loci that were commonly circularized were defined as any region in which 191 or more tumors had at least one circle present. Adjacent bins in commonly circularized genomic loci were merged using BEDtools, with any two bins within 1 kb of each other considered as coming from the same loci. Chromosome Y was removed from all samples for this analysis due to the presence of male and female patients and sex-specific cancers in the data set.

### 2.4. Identifying Circles with Genes and Circles without Genes

We grouped eccDNA based on gene content instead of by size, which departs from prior size-based nomenclature [3,4,5,6]. We instead refer to eccDNA regardless of size as circles, circles with genes, or circles without genes. Circles with genes were defined as any circle that contained a complete, non-fragmented functional GENCODE transcript. GENCODE GTF files for HG38 were downloaded from the GENCODE website (version 39): https://www.gencodegenes.org/ (accessed on 11 November 2021). Overlap between GENCODE genes and circular DNA was performed using BEDtools (version 2.3) [26]. Circles containing at least one whole gene were classified as circles with genes; however, any partial transcripts at a circle breakpoint were classified as genomic transcripts and not associated with a circle. Any circle containing part of a transcript or less was classified as a circle without genes.

### 2.5. Gene Expression Analysis

Upper quartile normalized FPKM RNAseq data were obtained through the Xena [23] UCSC server for 340 of the 355 tumors, representing 21 of the 22 cancer types (GBM had no samples with RNA-seq and circle data), with circle calls. We considered genes with a normalized FPKM value greater than zero expressed. We acquired pathways from the MSigDB (version 7.5.1), maintained by GSEA [27], and a modified Fisher’s exact test was used to determine the enrichment of expressed genes, as previously published [28]. Enrichment was performed for expressed genes on circles in individual tumors as well as for genes commonly found in circles from all tumors. Genes in circles for each individual tumor were ranked by their expression, and all genes with an expression greater than one standard deviation above the mean expression were used for pathway analysis for individual tumors. To define commonly expressed genes in circles, we ranked expressed genes by the number of tumors that contained each gene within a circle. Pathway enrichment analysis was performed on the genes in the ranked list that were two standard deviations above the mean. This process produced a list of list genes that were both expressed and commonly found in circles irrespective of tumor type.

### 2.6. Homology and Microhomology Analysis

Homology analysis was performed using default parameters with NCBI BLAST (https://blast.ncbi.nlm.nih.gov/Blast.cgi, (accessed on 10 January 2022)). These parameters look for a minimum length of homology of 28 bp and mismatches are allowed with a scoring penalty for longer lengths of homologous sequence. To search for homologous sequences between the beginning of a circle and its corresponding end, BED files for the BLAST procedure were generated to include 500 bp of circular DNA and 500 bp of genomic DNA from each end of the circle. Only circles larger than 1000 bp were used for this analysis to ensure no regions internal to the circle were overlapping. For each start and end coordinate pair, the start coordinate was used as the query sequence and the end coordinate was used as the database sequence. This procedure was repeated for each individual circle in each individual tumor. All alignments with an E value less than 1 were considered significant and used for further analysis. For controls, we used both a scrambled real circle control and the ends of the randomly generated circles. Sequences were scrambled using the R statistical suite (version 4.1.0). To identify microhomology, we considered the 100 bp of DNA on either side of each circle breakpoint and required circles larger than 200 bp for the analysis to ensure no internal overlap within the circle occurred. We adjusted the BLAST parameters to look for stretches of homology down to 4 bp and allowed for mismatches for longer lengths of homology, which is consistent with the length of microhomology observed for microhomology mediated repair (MHMR) processes in human cells [29,30,31,32]. We considered an E value less than 1 to be significant. All statistical analyses and figures generated were performed using the R statistical suite (version 4.1.0). An ANOVA with a post-hoc Tukey test was used to calculate significant differences between real circles, random circles, and a scrambled real circle control. To visualize the position of homology with respect to the breakpoint, we transferred the homology positions reported for each circle to either a ±500 number line for homology or a ±100 number line for microhomology, with the midpoint representing the circle breakpoint. The area within the coordinates of homology or microhomology was designated as “1”. These coordinates were summed for all circles and plotted as a line to show common areas where homology occurred with respect to the circle breakpoints.

### 2.7. Motif Enrichment

Motif enrichment was performed in R (version 4.1.0) using the Bioconductor package PWMEnrich (version 4.3, https://bioconductor.org/packages/release/bioc/html/PWMEnrich.html (accessed on 1 February 2022)) and the corresponding human version of the MotifDB (version 1.36, https://bioconductor.org/packages/release/bioc/html/MotifDb.html (accessed on 1 February 2022)). Default parameters were used for all steps of this analysis. We looked for enrichment at either end of circles, with an interval of ±250 bp, where each interval extended into the circle by 250 bp as well as beyond the circle into the genome by 250 bp. Circles larger than 500 bp were used to ensure no overlapping regions internal to the circles. A genome-wide database of human JASPER motifs [33] was downloaded from the UCSC genome browser [34] and used to confirm the positions of the motif enrichments with respect to the circle breakpoints. Any transcription factor with a predicted binding significance less than 0.001, determined by a statistical inference Fisher’s exact test [35], within the specified intervals was used. The position coordinates for significantly enriched motifs were transformed with respect to the circle breakpoint to fit on a −250 to +250 number line. Individual motifs were scored as a “1” at the bp positions of the motif and summed for all circles by each bp to visualize as a trace.

## 3. Results

### 3.1. Circles Are Commonly Generated at Specific Genomic Loci Independent of Cancer Type

**Figure 1:**
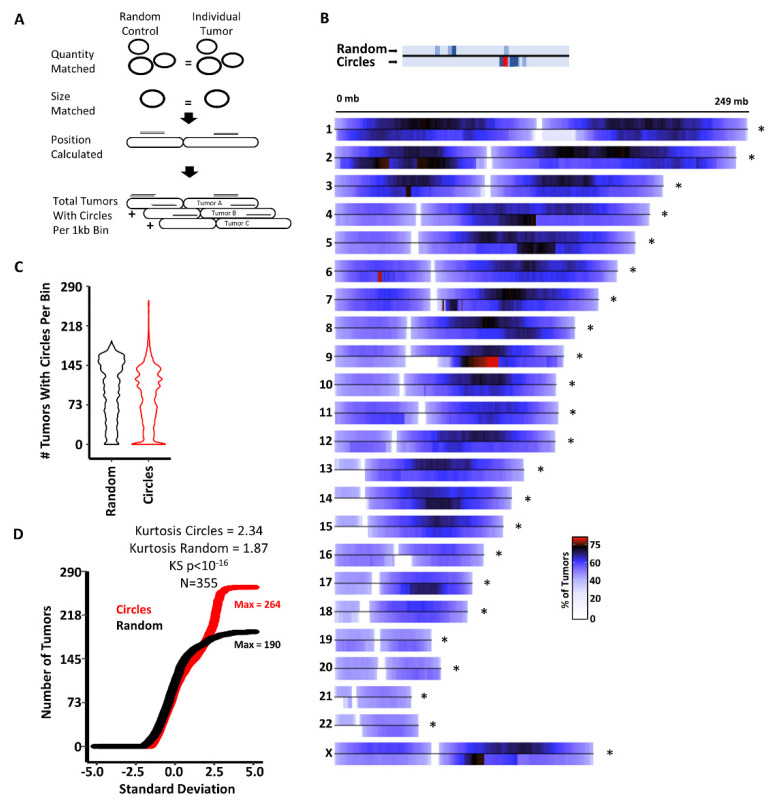
DNA circles are found at common loci irrespective of tumor type. (**A**) Schematic depicting the analysis for each of the 355 TCGA tumor samples. Random circles were constrained to non-centromere locations and forced to generate the same number of and size of real as each individual tumor. (**B**) Pan-cancer analysis of circles show many tumors have common genomic locations with circles. Top of each chromosome represents the distribution of random circle controls. Below the line represents true circles from TCGA samples. The transition from red to black indicates greater than 75% of samples with a circle in the bin. The coordinates for regions with circles in 191 or more tumors are reported in Table S1. (**C**) Distribution of the 3 million 1 kb bins with the number of samples with circles in each bin. (**D**) QQ analysis of the distribution of bins by number of samples. Significance of the overall distribution was assessed by KS test and the deviation of bins with high numbers of samples was assessed by Kurtosis. *p*-values less than 0.05 were considered significant. An * next to a chromosome indicates a *p*-values less than 10^−16^ by KS for that chromosome.

Previous analyses have demonstrated circles are distributed throughout the genome [3,10,14,16,19,24,36], but it is not clear if specific genomic regions are prone to forming circles. Furthermore, it is unclear if circles are generated from common locations regardless of tumor type, or if circle-producing regions are tumor-type specific. To investigate this, we asked if the distribution of circles in the genome is different than a randomly sampled controlled population of circles. We generated a pool of random control circles for each of the 355 tumors in our cohort (Figure 1A, see Methods). The random control circles were (1) not allowed to cross or overlap with centromeres and (2) had to result in the same size and quantity distribution of the circles from each individual tumor (Figure 1A).

To investigate if common locations in the genome produce circles across tumors, regardless of the number of circles at each location per tumor, we summed the number of tumors with at least one circle for each 1 kb bin in the genome and plotted these totals as a chromosome heatmap (see Methods). This revealed regions in the genome that commonly produce circles across the 22 cancer types analyzed (Figure 1B). The pattern of circle position between randomly generated circles and real circles was different genome wide and on each individual chromosome (KS test *p*-value < 10^−16^), indicating the locations of real circles are not random. Analysis of the distribution of bins by patient number exhibited an extended population with a high number of patients per bin (Figure 1C). Further analysis of the bin distribution by QQ plot demonstrated a clear deviation from the random circles for bins with high number of patients (Figure 1D, kurtosis = 2.34). To identify regions prone to circularization, we identified all bins with real circles with greater than 190 tumors (the maximum of tumors in bins from the random circles). This identified 52 regions that commonly generate circles irrespective of cancer type (Table S1). These data demonstrate circles occur non-randomly in the genome across cancer types, suggesting either a common mechanism may drive circle generation, or sequence-specific aspects of these regions are prone to circularization.

### 3.2. Characteristics of Circular DNAs from TCGA Samples

Previous analysis of tumor populations has indicated the existence of both circles with genes and circles without genes [3,8,10,14,16,17,19,24,36]. These different findings have led to a lack of consensus on the function of eccDNA. Understanding the differences between circles with genes and circles without genes, common to multiple cancer types, could identify the underlying biological function and generation of circular DNA. The mean number of circles per tumor was 521 (range = 63–8623); with a mean length of 2,230,272 bp ranging from 23 bp to 49,999,840 bp (Figure S1A). Circles were divided into two groups (see Methods): (1) those containing at least one functional transcript (referred to as circles with genes), and (2) circles that contained either part of a gene or no gene (referred to as circles without genes). The mean number of circles with genes per tumor was 103 (range = 24–493), with a mean length of 14,424,342 bp ranging from 152 bp of 49,999,840 bp in length (Figure S1B). The mean number of circles without genes per tumor was 428 (range = 38–8280), with a mean length of 3286 bp ranging from 23 bp to 30,057,358 bp (Figure S1C). The propensity for circles with genes to be longer than circles without genes is consistent with previous analyses of ecDNA and eccDNA [14].

### 3.3. Genes on Circles Are More Likely to Be Expressed and Are More Highly Expressed, than Other Genes in the Genome

Prior work has found specific genes, primarily oncogenes, on circles tend to be over expressed [16]. However, the full circle-associated transcriptome has yet to be characterized in a large cohort of human tumors from multiple cancer types. Notably, no method to date can definitively determine the difference between circle-transcribed RNA and chromosomal-transcribed RNA on a transcriptome-wide scale. Therefore, we use the term “circle-associated transcriptome” in reference to transcripts that originate from the same genomic regions contained within circular DNA. In this cohort of TCGA tumors, 340 tumors had RNA-seq available through TCGA and Xena, for 21 of the 22 cancer types (data were unavailable for GBM). To analyze the circle-associated transcriptome, we categorized all genes in the genome by those that were found within circles or as genes found elsewhere in the genome for each individual tumor. We performed this procedure for each tumor’s individual pool of circles and transcriptome.

**Figure 2:**
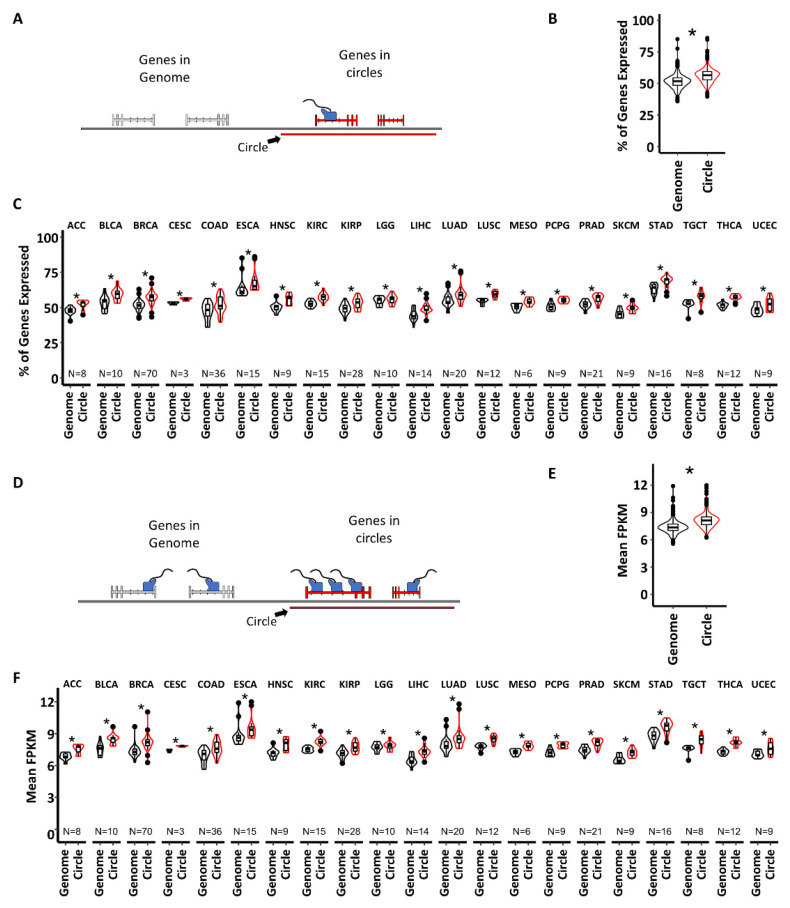
More genes in circles are expressed, and are more highly expressed, than genes located elsewhere in the genome. (**A**) Schematic showing how genes associated with circles were isolated. No genes that crossed a circle breakpoint were considered associated with a circle. (**B**) The pan-cancer distribution of the percent of genes expressed in the genome vs expression of genes associated with circles. (**C**) The distribution of the percentage of genes expressed in the genome vs genes associated with circles for individual cancer types. (**D**) Schematic showing how genes were categorized to determine the expression differences between genomic genes and genes associated with circles. (**E**) Pan-cancer analysis of mean gene expression for expressed genes in the genome vs gene associated circles. (**F**) Individual cancer analysis of mean gene expression for expressed genes in the genome vs genes associated with circles. Significance was determined by two tailed *t*-test, * indicates a *p*-value less than 0.05.

To determine if circle-associated-genes are more expressed, we first calculated the percentage of genes found in circles that were expressed, as well as for genes found elsewhere in the genome (Figure 2A). A higher percentage of genes found in circles are expressed compared to genes elsewhere in the genome (mean percent of genes expressed in circles = 56.51%; mean percentage of genes expressed in the genome = 51.81%; Fisher’s combined *p*-value < 10^−16^) (Figure 2B). A significantly higher percentage of genes on circles were also expressed more often in individual cancer types (Figure 2C). To determine if the circle-associated transcriptomes are more highly expressed than the genomically located transcriptome, we isolated all transcripts with an FPKM greater than zero and compared the mean expression of the circle-associated transcriptome and the genome transcriptome for each tumor (Figure 2D). Genes from the circle-associated transcriptome were more highly expressed than genes from elsewhere in the genome (mean FPKM of circle-associated transcriptome = 7.9, mean FPKM of genomic transcriptome = 7.1, Fisher’s combined of individual tumors *p* < 10^−16^) (Figure 2D,E). Genes from circles were also significantly more highly expressed in each individual cancer type (Figure 2F).

We then sought to identify enriched pathways to infer the molecular function of the circle-associated transcriptome. We took two approaches to test for enrichment. The first method considered the possibility that the circle-associated transcriptome for individual tumors may share enrichment for specific pathways, but the genes responsible for the enrichment may be different from cancer to cancer or tumor to tumor. The second method focused on the function and pathways of genes commonly found in circles irrespective of tumor or cancer type.

**Figure 3:**
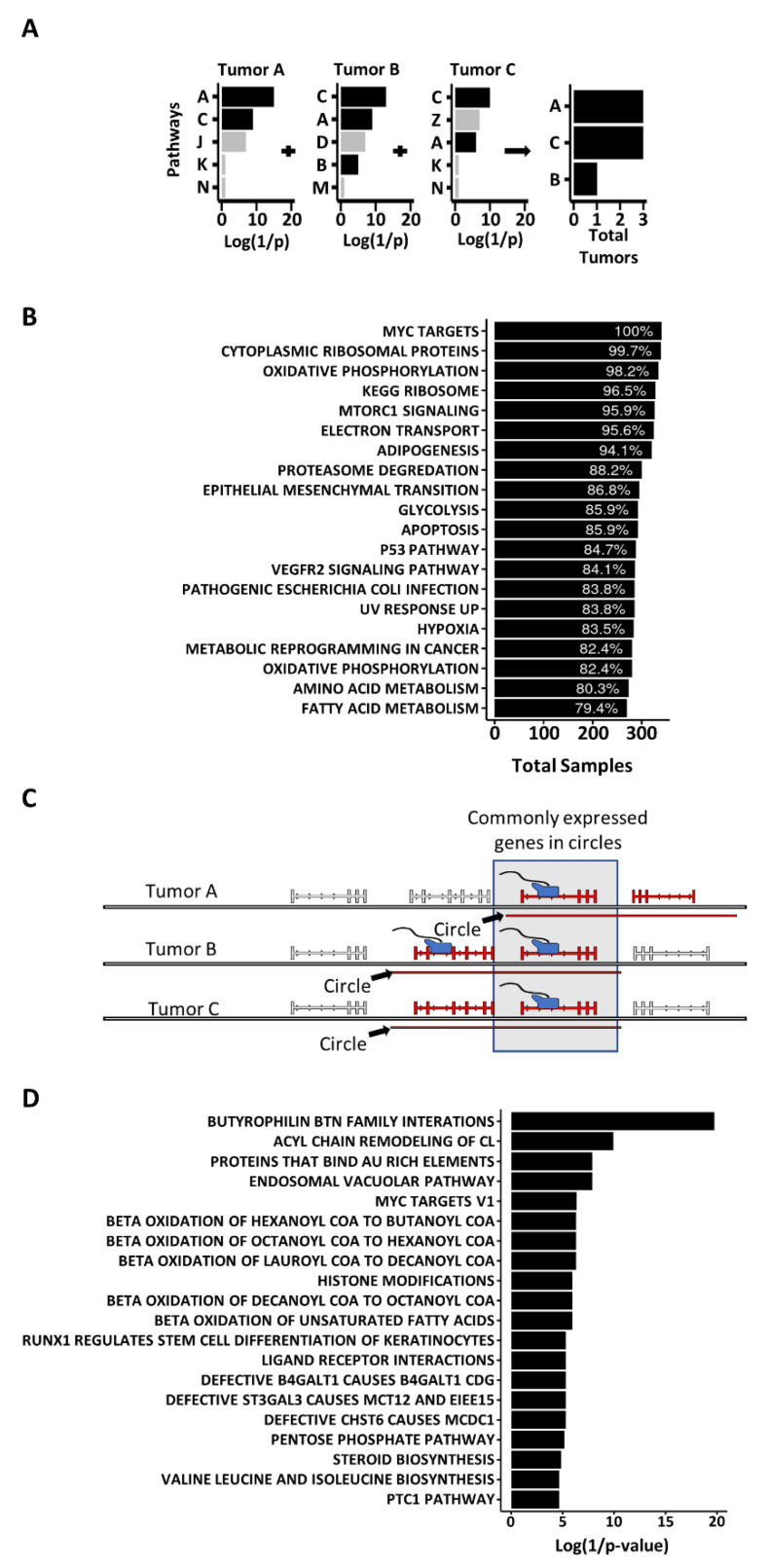
The circle-associated transcriptome is enriched with cancer-related pathways. (**A**) Schematic showing how commonly enriched pathways were defined. Pathway analysis was performed on the highest expressed genes in each tumor, defined as any gene with an FPKM value greater than one standard deviation above the mean. The common, significantly enriched pathways (in black) were then tallied across tumors and displayed as a waterfall plot. (**B**) Commonly enriched pathways in the circle-associated transcriptome. The percent of tumors with a significant enrichment of a specific pathway is indicated in white lettering inside each bar, while the length of each bar indicates the absolute number of samples with a significant enrichment (see Methods). (**C**) Schematic showing the isolation of genes commonly found in circles, prior to pathway enrichment. Expressed genes found in circles were isolated for each individual tumor. The number of tumors that expressed a gene located in a circle was totaled for all genes. (**D**) Pathways common to genes found in circles in greater than 50% of tumors. The length of the bar is the log10(1/*p*-value). Significance for enrichment was determined using a modified Fisher’s exact text with a *p*-values less than 0.05 considered significant.

To identify commonly enriched pathways in all tumors, we calculated pathway enrichments for highly expressed circle-associated genes in each individual tumor using the MSigDB’s hallmark and canonical pathway gene set collections (see Methods). The resulting pathways were filtered by significant enrichment, defined as a Fisher’s exact *p*-value less than 0.05, and then totaled the number of tumors with significant enrichment and displayed this as a waterfall plot depicting the number and percentage of samples with enrichment for each pathway (Figure 3A). Enriched pathways present in the circle-associated transcriptome of most samples were metabolic and well-known cancer-related pathways; specifically, the MSigDB hallmark pathway for MYC targets was enriched in every tumor (Figure 3B). Other notable cancer-related pathways enriched in most tumors were P53, MTORC1 signaling, VEGF signaling, and the epithelial to mesenchymal transition (EMT). These findings agree with prior work suggesting that genes in circles from cancer samples may be related to oncogenesis [8,16,17,18,19]. However, this analysis provides further clarification that this is not cancer type-specific and not restricted to oncogenic driver genes. The enrichment of metabolic pathways was similarly broad in scope but was most enriched in oxidative phosphorylation and metabolic reprogramming related to cancer. The enrichment of P53 and response to UV pathways suggests DNA damage and DNA repair mechanisms are enriched in the circle-associated transcriptome as well. A complete list of pathways enriched in more than 50% of tumors can be found in Table S2.

To investigate the function of genes commonly found in circles regardless of cancer type, we generated a list of expressed genes commonly found in circles in 21 tumor types by totaling the instances in which a functional transcript was found in a circle per tumor (Figure 3C) (see Methods). Using the MSigDB canonical and hallmark pathway gene set collections, we determined the pathway enrichment of genes commonly found in the circle-associated transcriptome (Figure 3D). We found these genes are enriched in mechanisms of immune response mediation by BTN interactions, genes involved in fatty acid beta oxidation, and several pathways related to protein glycosylation. Additionally, this analysis identified enrichment of the pentose phosphate pathway, which is responsible for nucleotide synthesis and NADPH production. We also identified several pathways related to mechanisms of oncogenesis, including ligand–receptor interactions, genes targeted by MYC, stem cell maintenance through RUNX1/CBFB, and cell cycle regulation mediated by Hedgehog signaling (the PTC1 pathway). A complete list of significantly enriched pathways can be found in Table S3.

Analysis of the MSigDB genomic position gene set collection confirmed the list of genes commonly found expressed in circles are also enriched for the same regions identified as commonly producing circles in high numbers of samples (Figure S2A in comparison to Figure 1B and Table S1). Looking at the individual genes in this analysis, we noticed an abundance of genes from families of highly homologous genes. This prompted us to investigate the enrichment of homologous gene families. Using a database of homologous gene families [37], we identified several families of zinc-finger proteins, as well as families of proteins related to the immune system (BTN and HLA families), folate receptors, TLE transcription co-repressor families, among several other families (Figure S2B). The enrichment of multiple families of homologous genes suggests homology may play a role in the generation, maintenance, or selection of circular DNA in cancer.

### 3.4. Some Circles Are Self-Homologous

**Figure 4:**
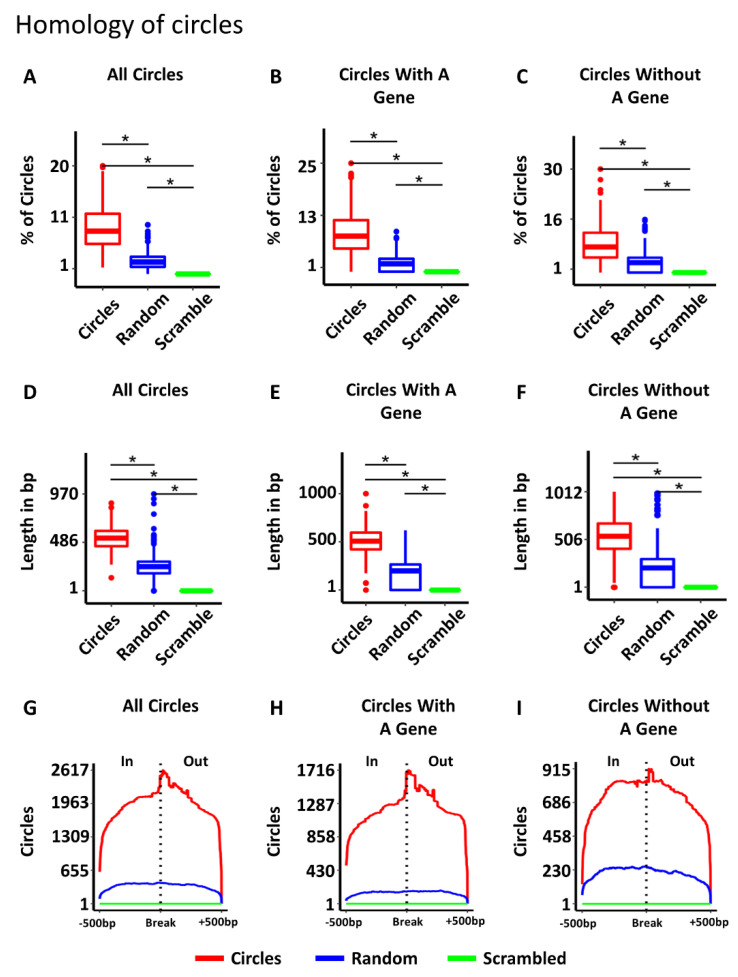
The breakpoints of circular DNA exhibit self-homology. (**A**) Percent of circles with self-homology compared to random and scrambled controls. (**B**) Percent of circles with genes with self-homology compared to random and scrambled controls. (**C**) Percent of circles without genes with self-homology compared to random and scrambled controls. (**D**) Length of homology in bp for circles compared to random and scrambled controls. (**E**) Length of homology in bp for circles with genes compared to random and scrambled controls. (**F**) Length of homology in bp for circles without genes compared to random and scrambled controls. (**G**) Homology is enriched near the circle breakpoint in all circles. (**H**) Homology is enriched near the circle breakpoint in circles with genes. (**I**) Homology is enriched near the circle breakpoint in circles without genes. In = region inside the circle, out = region outside the circle. Significance was determined using an ANOVA, with a post-hoc Tukey test. * indicates *p*-values less than 0.05.

One of the key questions about circular DNA in cancer is how the circles are produced. Several mechanisms have been proposed including: homologous recombination (HR) [3,7,8,9], ligation of fragmented apoptotic DNA [10], replication slippage [11], byproducts of DNA double strand break (DSB) repair [3,5,7,8,9], and episome-polymerization [12,13]. The enrichment of homologous gene families on circles regardless of tumor type (Figure S2B) is consistent with a role for homology in the production of circular DNA. To determine if HR might play a role in the production of circular DNA, we isolated circles greater than 1000 bp in length and searched for homology within 500 bp of the two ends of individual circles (See Methods). A total of 8.63% of all circles had homology between the two ends, which was significantly more than the ends of random circles (2.39% with homology) or scrambling the actual circle ends as controls (0% with homology) (ANOVA = *p*-value < 10^−16^; Tukey test post-hoc: circle-to-random *p*-value < 10^−16^, circle-to-scrambled *p*-value < 10^−16^) (Figure 4A). For circles with homology, the length of homology was also significantly longer than random controls (Figure 4D). Circles had an average length of homology of 528 bp, while random controls (the 2.39% that had homology) had a mean homology length of 238 bp (ANOVA = *p*-value < 10^−16^; Tukey test post-hoc = *p*-value < 10^−16^). To see where the homology occurred with respect to the circle ends, we plotted the position of homology ±500 bp from the breakpoint for each circle, random control, and scrambled control, and displayed the summed trace (Figure 4G). The peak of homology in the circles is clearly centered over the breakpoint, while the homology found in either of the controls was dispersed across the whole region. The circles containing genes and circles without genes also had significantly more homologous circles (Figure 4B,C), longer stretches of homology (Figure 4E,F), and homology enriched over circle breakpoints compared to random controls and scrambled controls (Figure 4H,I). The higher percentage of circles with homology, the longer length of homology, and the position of homology at the circle breakpoint regardless of the gene content of the circles supports a role for HR in the generation of some circles.

### 3.5. Most Circles Are Self-Microhomologous

**Figure 5:**
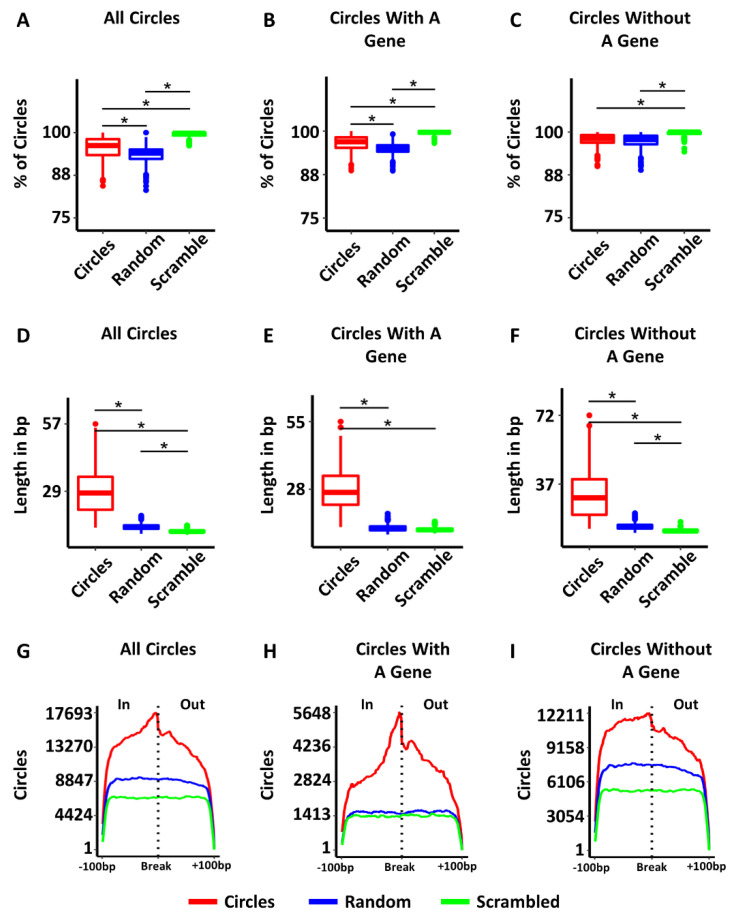
Circles are self-microhomologous. (**A**) Percent of circles with self-microhomology compared to random and scrambled controls. (**B**) Percent of circles with genes with self-microhomology compared to random and scrambled controls. (**C**) Percent of circles without genes with self-microhomology compared to random and scrambled controls. (**D**) Length of microhomology in bp for circles compared to random and scrambled controls. (**E**) Length of microhomology for circles with genes compared to random and scrambled controls. (**F**) Length of without genes compared to random and scrambled controls. (**G**) Traces showing the position of microhomology with respect to the circle breakpoint for all circles, random and scrambled controls. (**H**) Traces showing the position of microhomology with respect to the circle breakpoint for circles with genes, random and scrambled controls. (**I**) Traces showing the position of microhomology with respect to the circle breakpoint for all circles without genes, random and scrambled controls. Significance was calculated by ANOVA, with a post-hoc Tukey test. * indicates *p*-values less than 0.05.

Microhomology mediated repair (MHMR) is another common group of mechanisms for repairing DSBs, that includes, among others, microhomology-mediated synthesis-dependent strand annealing (MM-SDSA) and microhomology-mediated end joining (MMEJ) [29,30,31,32,38]. These mechanisms are known to be active in many human cancers [29,30,31]. This method of end joining relies on smaller intervals of 4–20 bp of homology in human cells, though the same mechanism is known to function with as few as 1 bp in other species [29,30,31]. Considering approximately 9% of tumors exhibited extended lengths of homology associated with HR, we altered our homology parameters to identify lengths of microhomology down to 4 bp to investigate the extent of microhomology between circle ends. With such small lengths of homology, the circles (mean = 96.52%), random controls (mean = 94.97%) and scrambled controls (mean = 99.53%) all exhibited microhomology in the majority of all samples (ANOVA = *p*-value < 10^−16^; Tukey test post-hoc: circle-to-random *p*-value < 10^−16^, circle-to-scrambled *p*-value < 10^−16^) (Figure 5A). The length of the microhomology in circles was significantly greater in length (mean = 28.95 bp) than random controls (mean = 14.04 bp) or scrambled controls (mean = 12.28 bp) (ANOVA = *p*-value < 10^−16^; Tukey test post-hoc: circle-to-random *p*-value < 10^−16^, circle-to-scrambled *p*-value < 10^−16^) (Figure 5D). Plotting the traces of microhomology revealed an enrichment of microhomology centered on the breakpoint in circles, and the shorter lengths of microhomology found in both random and scrambled controls were dispersed throughout the region (Figure 5G). Circles with genes and circles without genes also exhibited longer lengths of microhomology (Figure 5E,F) and enrichment of microhomology around the circle breakpoint (Figure 5H,I). The higher number of circles with microhomology suggests MHMR is likely significantly involved in circle generation.

### 3.6. Circle Ends Are Enriched in Transcription Factor DNA Binding Motifs

**Figure 6:**
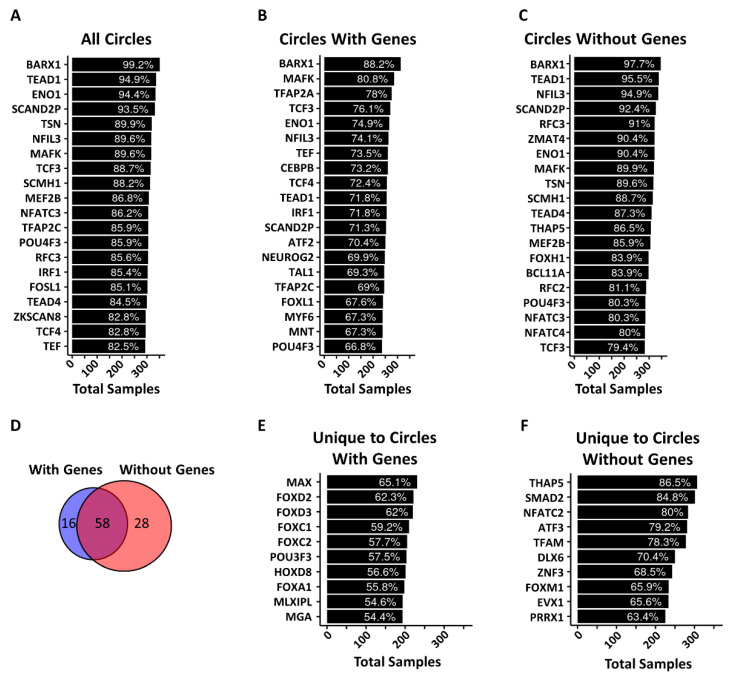
DNA binding motifs are enriched at circle breakpoints. (**A**) Most commonly enriched DNA binding motifs found within ±250 bp of a circle breakpoint for individual tumors. The white lettering shows the percent of tumors with enrichment of the motif while the length of the bar is the absolute number of tumors. (**B**) Most commonly enriched DNA binding motifs found within ±250 bp of the breakpoint of circles with genes for individual tumors. The white lettering shows the percent of tumors with enrichment of the motif while the length of the bar is the absolute number of tumors. (**C**) Most commonly enriched DNA binding motifs found within ±250 bp of the breakpoint of circles without a gene for individual tumors. The white lettering shows the percent of tumors with enrichment of the motif while the length of the bar is the absolute number of tumors. (**D**) Venn diagram depicting the overlap of the total number of enriched motifs found in at least 50% of tumors for circles with genes and circles without genes. (**E**) Ten most commonly enriched DNA binding motifs in circles with genes that are not enriched in circles without genes. (**F**) Ten most commonly enriched DNA binding motifs in circles without genes that are not enriched in circles with genes. All values of significance were calculated in the PWMEnrich R package and are based on a simulated modified Fisher’s exact test. Values less than 0.001 were considered significant for this analysis. The complete list of factors with motif enrichments can be found in Tables S4 and S5.

Based on our finding that circles with genes originate from regions in the genome with higher gene expression, we hypothesized that transcription factors may play a role in circle formation and thus we investigated the presence of DNA binding motifs near circle ends. We used the PWMEnrich Bioconductor package to look for enriched DNA binding motifs within the 250 bp on either side of each circle breakpoint for circles longer than 500 bp. To identify motifs common to circles regardless of tumor type, we totaled the number of instances a motif was significantly enriched at circle breakpoints in all 355 tumors (Figure 6A). We repeated the same analysis for circles with genes (Figure 6B), and circles without genes (Figure 6C). A complete list of all factors with enriched motifs in greater than 50% of tumors can be found in Tables S4 and S5. Of the transcription factors with motifs identified, 58 were common between circles with genes and circles without genes (Figure 6D). However, we identified 16 motifs unique to circles with genes and 28 motifs unique to circles without genes (Figure 6D–F). Finally, we verified the expression of each of these factors in each tumor type to see if these factors were expressed in the tumors in our cohort (Figure S3). Most of these factors were robustly expressed in all tumor types which means these factors are likely present and could bind these motifs.

Of the motifs most enriched at circle breakpoints, or differentially enriched between circles with genes or without genes, 21 of these motifs are known TFs and are well annotated in the JASPER TF database, which have genome-wide binding sites available through the UCSC genome browser. To see where these motifs are located with respect to the circle ends, we plotted the motif positions ±250 bp with respect to the circle breakpoints (Figures S4–S7). The motif positions were organized by pattern: (1) forming a distinct peak inside of the breakpoint within the circle (In, Figure S4), forming a distinct peak outside of the circle (Out, Figure S5), forming distinct peaks to either side of the breakpoint (Figure S6), and dispersed over the whole region (Figure S7). Notably, two motifs did abut the breakpoint: BARX1 (Figure S4A) and FOXL1 (Figure S4B). Our results suggest that theses motifs and the factors that bind them may facilitate circle production, maintenance, or selection.

## 4. Discussion

The existence of extrachromosomal circular DNA is an established feature of cancer [8,16,17,18,19], however many fundamental questions remain regarding the origin, function, maintenance, or selection of these molecules. In this study, we provide evidence to shed light on these important questions. Our analysis of circles from TCGA tumors demonstrates specific genomic regions are more likely to produce circles regardless of tumor type. The genes found in these circles are more likely to be expressed, and are expressed to a higher level, than genes not found in circles from the same tumors. This suggests that circles are either more likely to arise from transcriptionally active regions of the genome or that circles containing genes, likely important to cancers, are selected for and maintained in tumors.

Much of the work investigating cancer specific circles has focused on the presence of known oncogenes in circles and has found these genes are often overexpressed [8,16,17,18,19], but an analysis of the circle-associated transcriptome in cancer has yet to be reported. We show that more of the genes on circles are expressed, and are more highly expressed, than genes elsewhere in the genome. However, the data we used for this study did not allow us to determine if the increase in expression is due to expression from the circle or is chromosomal in origin. The functional categories of these genes are enriched in mechanisms of oncogenesis, metabolism, and immune regulation, which are commonly enriched pathways in transcriptome-wide analyses of cancer [39,40,41]. Identifying common pathways, regardless of tumor type, suggests these circles may have a common function in cancer, or are related to common oncogenic, metabolic, or immune processes in cancer. However, it remains unclear if these pathways are related to mechanisms of circle biogenesis, regulation or selection, which is an area for future investigation.

How extrachromosomal circles are formed is an unresolved aspect of circular DNA in cancer. Many cancers lose function in DNA damage response (DDR) pathways, resulting in a marked increase in genomic instability and accumulation of damaged DNA [29,30,31,38,42,43,44]. The progressive accumulation of DNA damage in cancer could provide a source of DNA for circle formation through the ligation of DSBs and closure of fragments by DNA repair pathways [3,4,5,24,25,29,30,31,38]. Many DNA repair pathways rely on various forms of homology to join DSBs including HR, MHMR, MMEJ, and MM-SSA [29,30,31,32,38]. Questions regarding the relationship of DNA damage processes and DSB repair with respect to circle formation remain unanswered and are a critical part of understanding the fundamental nature of these molecules.

In support of a role for homology in the biogenesis of circles, the data shown here demonstrate circle breakpoints from TCGA tumors are self-homologous. Most of this homology is microhomology and suggests MHMR pathways may be the primary class of mechanisms for circle end joining. MMEJ is a related pathway to non-homologous end-joining (NHEJ), that relies on a small degree of homology to join double stand breaks, usually between 4–20 bp in human cell lines and cancer [29,30,31]. Prior work has demonstrated that over 75% of breaks are repaired with some form of NHEJ [44], and that MMEJ is highly relied on in the context of cancer due to a loss of HR [29,31]. Given that 94% of circles were self-microhomologous in our cohort compared to only 9% of circles with self-homology, this suggests MHMR may play a significant role in circle end-joining across cancer.

Surprisingly, we identified the enrichment of specific DNA binding motifs at the ends of circles. Most of the motifs identified are from transcription factors expressed in most tumors regardless of cancer type. This implies these motifs are likely to be bound in these cell types and that these binding sites may play a role in the production, maintenance, or selection of the circular DNA. It is possible the factors found here promote the transcription of these regions and may cause double strand DNA breaks (DSBs). In support of this, there is an increase in DSBs at actively transcribed genes to relieve torsional strain in DNA and allow for efficient transcription [42,44,45], and thus the formation of circles at these sites might be a function of normal transcription associated DSBs in combination with erroneous DSB repair. Alternatively, the factors may function to alter chromatin accessibility at these loci to promote DSB repair and HR or MHMR. Additionally, we identified binding motifs for replication factor C (RFC2 and RFC3) perhaps suggesting DNA replication or DNA rereplication could play a role in circle biogenesis. While the data available from TCGA do not allow us to identify the precise mechanism for circle formation, this analysis does provide important insights for future experimental validation to determine the importance of TF binding sites to circular DNA biology.

Prior studies have reported mixed results on whether circles are generated randomly or occur in common areas of the genome, and whether these molecules serve a biological function or are a biproduct secondary to another cellular event [8,10,16,17,18,19,21]. Many of these differences can be attributed to the method used to study circles, in which circles isolated by circle-seq tend to be smaller and dispersed throughout the genome, while circles identified from next generation sequencing data include robust detection of larger circles that contain genes. Comparing the circles based on gene content allowed determination of similarities and differences between smaller circles that lack a whole functional gene and larger circles that contain functional genes. Demonstrating similar degrees of homology and microhomology suggests circles, regardless of content, have similar mechanisms of formation. The identification of evidence consistent with common mechanisms of circle formation suggests circles, small and large, may have common origins.

## 5. Conclusions

In this study we demonstrate circular DNAs originate from common genomic locations irrespective of tumor type. The genes contained in circular DNA are more likely to be expressed and are more highly expressed than genes elsewhere in the genome. We also show eccDNA ends are mainly self-microhomologous, with a minority of ends having homology. Finally, we show the enrichment of motifs from expressed DNA binding proteins at eccDNA ends. Our results support a model for homology-related processes in circle biogenesis. 

## Data Availability

Circular eccDNA identification was previously published [22] and can be obtained from http://genome.bioch.virginia.edu/TCGA-ATACSEQ-ECCDNA/ (accessed on 1 August 2021). All clinical and molecular TCGA data can be accessed through the Xena UCSC genome browser [23]: http://xena.ucsc.edu/ (accessed on 17 December 2021). Scripts for analysis can be downloaded from the Black lab GitHub link: https://github.com/Black-Lab-UCDenver/CircleAnalysis (made available on 11 April 2022).

## Supplementary Materials

The following are available online at https://www.mdpi.com/article/10.3390/cancers14092310/s1, Figure S1: Size distributions of extrachromosomal circular DNA, Figure S2: Specific chromosomal locations and homologous gene families are enriched for genes commonly found in circles, Figure S3: Transcription factors with DNA motifs at circle breakpoints are expressed in tumors, Figure S4: Position of motifs enriched inside the circle adjacent to the breakpoint, Figure S5: Positions of motifs enriched outside the circle adjacent to the breakpoint, Figure S6: Positions of motifs enriched on both sides of the breakpoint, Figure S7: Positions of motifs enriched surrounding circle ends. Table S1: Commonly circularized loci. This file contains all regions in which 191 or more of the samples had at least one circle present. Successive 1 kb bins exceeding representation by 191 samples were concatenated. The data are in a BED file format with header, and includes the chromosome, start, and stop positions in hg38 coordinates. Table S2: Complete list of pathways enriched from samples. Pathway enrichment was performed for the circle-associated transcriptome in individual tumors. For each pathway that was enriched in at least 50% of tumors the percentage of tumors with enrichment are shown. Table S3: Complete list of pathways enriched in genes common to circles. Pathways of commonly circularized genes. Pathway analysis was performed on commonly circularized genes. All pathways that reached significance are shown. Table S4: Motifs enriched at ends of circles with genes. DNA motif enrichment analysis was performed for individual tumors on the ends of circles with genes. This file shows all motifs found enriched in more than 50% of tumors. Table S5: Motifs enriched at ends of circles without genes. DNA motif enrichment analysis was performed for individual tumors on the ends of circles without genes. This file shows all motifs found enriched in more than 50% of tumors.

## References

[1] Hotta Y., Bassel A. (1965). Molecular size and circularity of DNA in cells of mammals and higher plants. Proc. Natl. Acad. Sci. USA.

[2] Radloff R., Bauer W., Vinograd J. (1967). A dye-buoyant-density method for the detection and isolation of closed circular duplex DNA: The closed circular DNA in HeLa cells. Proc. Natl. Acad. Sci. USA.

[3] Zuo S., Yi Y., Wang C., Li X., Zhou M., Peng Q., Zhou J., Yang Y., He Q. (2022). Extrachromosomal Circular DNA (eccDNA): From Chaos to Function. Front. Cell Dev. Biol..

[4] Ott C.J. (2020). Circles with a Point: New Insights into Oncogenic Extrachromosomal DNA. Cancer Cell.

[5] Wang T., Zhang H., Zhou Y., Shi J. (2021). Extrachromosomal circular DNA: A new potential role in cancer progression. J. Transl. Med..

[6] Yan Y., Guo G., Huang J., Gao M., Zhu Q., Zeng S., Gong Z., Xu Z. (2020). Current understanding of extrachromosomal circular DNA in cancer pathogenesis and therapeutic resistance. J. Hematol. Oncol..

[7] Møller H.D., Parsons L., Jørgensen T.S., Botstein D., Regenberg B. (2015). Extrachromosomal circular DNA is common in yeast. Proc. Natl. Acad. Sci. USA.

[8] Kim H., Nguyen N.-P., Turner K., Wu S., Gujar A.D., Luebeck J., Liu J., Deshpande V., Rajkumar U., Namburi S. (2020). Extrachromosomal DNA is associated with oncogene amplification and poor outcome across multiple cancers. Nat. Genet..

[9] Mukherjee K., Storici F. (2012). A Mechanism of Gene Amplification Driven by Small DNA Fragments. PLoS Genet..

[10] Wang Y., Wang M., Djekidel M.N., Chen H., Liu D., Alt F.W., Zhang Y. (2021). eccDNAs are apoptotic products with high innate immunostimulatory activity. Nature.

[11] Dillon L.W., Kumar P., Shibata Y., Wang Y.-H., Willcox S., Griffith J.D., Pommier Y., Takeda S., Dutta A. (2015). Production of Extrachromosomal MicroDNAs Is Linked to Mismatch Repair Pathways and Transcriptional Activity. Cell Rep..

[12] Carroll S.M., De Rose M., Gaudray P., Moore C.M., VanDevanter D., Von Hoff D.D., Wahl G.M. (1988). Double minute chromosomes can be produced from precursors derived from a chromosomal deletion. Mol. Cell. Biol..

[13] Wahl G.M., Vincent B.R.D.S., De Rose M. (1984). Effect of chromosomal position on amplification of transfected genes in animal cells. Nature.

[14] Koche R.P., Rodriguez-Fos E., Helmsauer K., Burkert M., MacArthur I.C., Maag J., Chamorro R., Munoz-Perez N., Puiggròs M., Garcia H.D. (2019). Extrachromosomal circular DNA drives oncogenic genome remodeling in neuroblastoma. Nat. Genet..

[15] Von Hoff D.D., McGill J.R., Forseth B.J., Davidson K.K., Bradley T.P., Van Devanter D.R., Wahl G.M. (1992). Elimination of extrachromosomally amplified MYC genes from human tumor cells reduces their tumorigenicity. Proc. Natl. Acad. Sci. USA.

[16] Turner K.M., Deshpande V., Beyter D., Koga T., Rusert J., Lee C., Li B., Arden K., Ren B., Nathanson D.A. (2017). Extrachromosomal oncogene amplification drives tumour evolution and genetic heterogeneity. Nature.

[17] Morton A., Dogan-Artun N., Faber Z., MacLeod G., Bartels C.F., Piazza M., Allan K.C., Mack S.C., Wang X., Gimple R.C. (2019). Functional Enhancers Shape Extrachromosomal Oncogene Amplifications. Cell.

[18] Wu S., Turner K.M., Nguyen N.-P., Raviram R., Erb M., Santini J., Luebeck J., Rajkumar U., Diao Y., Li B. (2019). Circular ecDNA promotes accessible chromatin and high oncogene expression. Nature.

[19] Weiser A., Hung K., Chang H. (2022). Oncogene Convergence in Extrachromosomal DNA Hubs. Cancer Discov..

[20] Nathanson D.A., Gini B., Mottahedeh J., Visnyei K., Koga T., Gomez G., Eskin A., Hwang K., Wang J., Masui K. (2014). Targeted Therapy Resistance Mediated by Dynamic Regulation of Extrachromosomal Mutant EGFR DNA. Science.

[21] Møller H.D., Mohiyuddin M., Prada-Luengo I., Sailani M.R., Halling J.F., Plomgaard P., Maretty L., Hansen A.J., Snyder M.P., Pilegaard H. (2018). Circular DNA elements of chromosomal origin are common in healthy human somatic tissue. Nat. Commun..

[22] Kumar P., Kiran S., Saha S., Su Z., Paulsen T., Chatrath A., Shibata Y., Shibata E., Dutta A. (2020). ATAC-Seq Identifies Thousands of Extrachromosomal Circular DNA in Cancer and Cell Lines. Sci. Adv..

[23] Goldman M.J., Craft B., Hastie M., Repečka K., McDade F., Kamath A., Banerjee A., Luo Y., Rogers D., Brooks A.N. (2020). Visualizing and interpreting cancer genomics data via the Xena platform. Nat. Biotechnol..

[24] Wu S., Bafna V., Chang H.Y., Mischel P.S. (2022). Extrachromosomal DNA: An Emerging Hallmark in Human Cancer. Annu. Rev. Pathol. Mech. Dis..

[25] Bailey C., Shoura M., Mischel P., Swanton C. (2020). Extrachromosomal DNA—Relieving heredity constraints, accelerating tumour evolution. Ann. Oncol..

[26] Quinlan A.R., Hall I.M. (2010). BEDTools: A flexible suite of utilities for comparing genomic features. Bioinformatics.

[27] Subramanian A., Tamayo P., Mootha V.K., Mukherjee S., Ebert B.L., Gillette M.A., Paulovich A., Pomeroy S.L., Golub T.R., Lander E.S. (2005). Gene set enrichment analysis: A knowledge-based approach for interpreting genome-wide expression profiles. Proc. Natl. Acad. Sci. USA.

[28] Jiao X., Sherman B.T., Huang D.W., Stephens R., Baseler M.W., Lane H.C., Lempicki R.A. (2012). DAVID-WS: A stateful web service to facilitate gene/protein list analysis. Bioinformatics.

[29] Seol J.-H., Shim E.Y., Lee S.E. (2017). Microhomology-mediated end joining: Good, bad and ugly. Mutat. Res. Mol. Mech. Mutagen..

[30] Wang H., Xu X. (2017). Microhomology-mediated end joining: New players join the team. Cell Biosci..

[31] Sfeir A., Symington L.S. (2015). Microhomology-Mediated End Joining: A Back-up Survival Mechanism or Dedicated Pathway?. Trends Biochem. Sci..

[32] Villarreal D.D., Lee K., Deem A., Shim E.Y., Malkova A., Lee S.E. (2012). Microhomology Directs Diverse DNA Break Repair Pathways and Chromosomal Translocations. PLoS Genet..

[33] A Castro-Mondragon J., Riudavets-Puig R., Rauluseviciute I., Lemma R.B., Turchi L., Blanc-Mathieu R., Lucas J., Boddie P., Khan A., Pérez N.M. (2021). JASPAR 2022: The 9th release of the open-access database of transcription factor binding profiles. Nucleic Acids Res..

[34] Kent W.J., Sugnet C.W., Furey T.S., Roskin K.M., Pringle T.H., Zahler A.M., Haussler D. (2002). The Human Genome Browser at UCSC. Genome Res..

[35] Sheffield N.C., Bock C. (2015). LOLA: Enrichment analysis for genomic region sets and regulatory elements in R and Bioconductor. Bioinformatics.

[36] Ling X., Han Y., Meng J., Zhong B., Chen J., Zhang H., Qin J., Pang J., Liu L. (2021). Small extrachromosomal circular DNA (eccDNA): Major functions in evolution and cancer. Mol. Cancer.

[37] Ouedraogo M., Bettembourg C., Bretaudeau A., Sallou O., Diot C., Demeure O., Lecerf F. (2012). The Duplicated Genes Database: Identification and Functional Annotation of Co-Localised Duplicated Genes across Genomes. PLoS ONE.

[38] Patterson-Fortin J., D’Andrea A.D. (2020). Exploiting the Microhomology-Mediated End-Joining Pathway in Cancer Therapy. Cancer Res..

[39] Uhlén M., Zhang C., Lee S., Sjöstedt E., Fagerberg L., Bidkhori G., Benfeitas R., Arif M., Liu Z., Edfors F. (2017). A pathology atlas of the human cancer transcriptome. Science.

[40] Rhodes D.R., Chinnaiyan A.M. (2005). Integrative analysis of the cancer transcriptome. Nat. Genet..

[41] Cieślik M., Chinnaiyan A.M. (2017). Cancer transcriptome profiling at the juncture of clinical translation. Nat. Rev. Genet..

[42] Marnef A., Cohen S., Legube G. (2017). Transcription-Coupled DNA Double-Strand Break Repair: Active Genes Need Special Care. J. Mol. Biol..

[43] Davis A.J., Chen D.J. (2013). DNA double strand break repair via non-homologous end-joining. Transl. Cancer Res..

[44] Ui A., Chiba N., Yasui A. (2020). Relationship among DNA double-strand break (DSB), DSB repair, and transcription prevents genome instability and cancer. Cancer Sci..

[45] Schwer B., Wei P.-C., Chang A.N., Kao J., Du Z., Meyers R.M., Alt F.W. (2016). Transcription-associated processes cause DNA double-strand breaks and translocations in neural stem/progenitor cells. Proc. Natl. Acad. Sci. USA.

