# Supplementary material for: Extrachromosomal Circular DNA from TCGA Tumors Is Generated from Common Genomic Loci, Is Characterized by Self-Homology and DNA Motifs near Circle Breakpoints
Source: Cancers (Basel). 2022 May 6;14(9):2310. doi: 10.3390/cancers14092310 (PMC9101409; doi:10.3390/cancers14092310)
Supplement: Supplementary file 1 [file cancers-14-02310-s001.zip › Tatman Supplemental Figures.pdf]

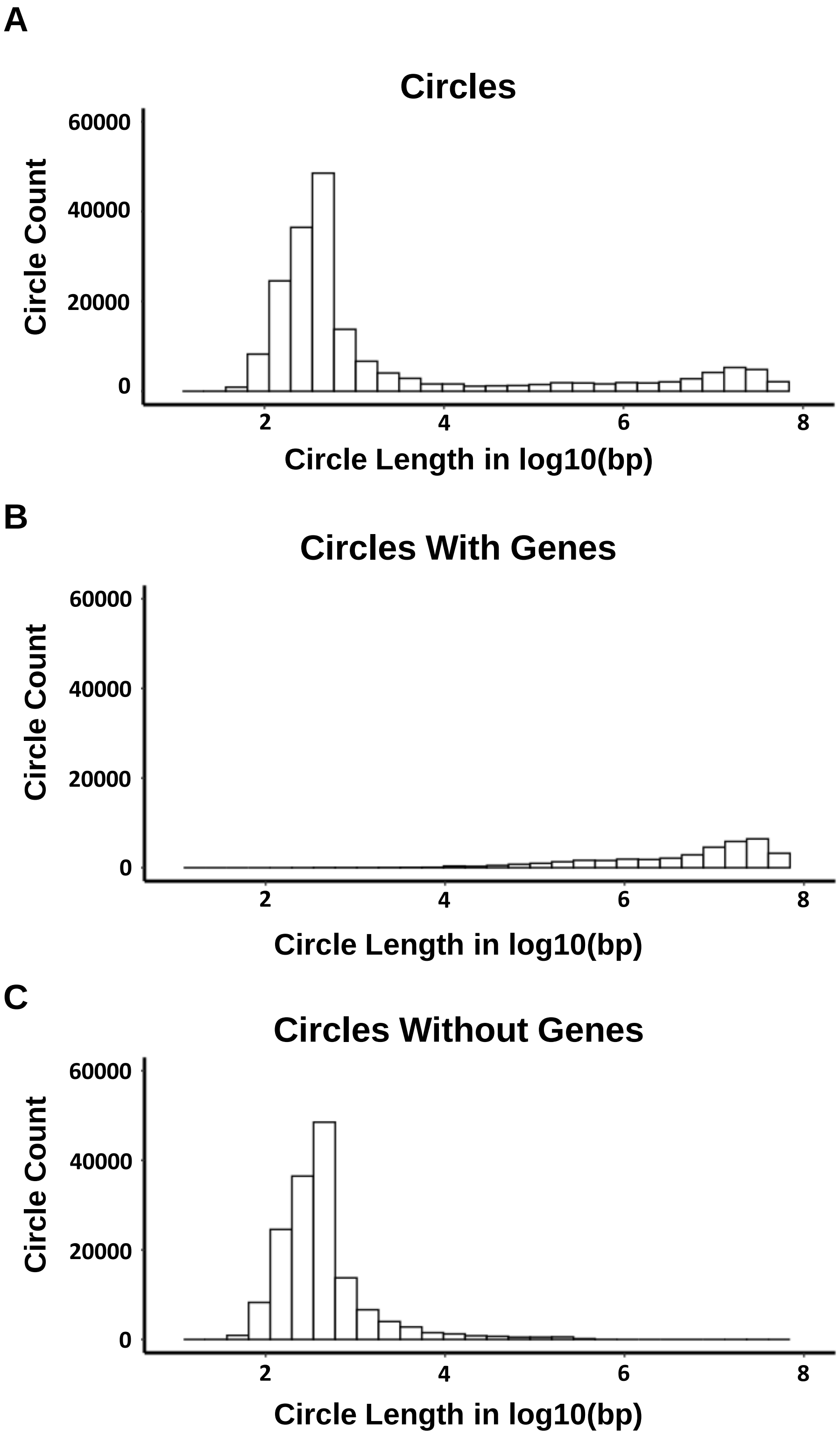

Supplemental Figure S1. Size distributions of extrachromosomal circular DNA. (**A**) Distribution of Log10 adjusted circle length in basepairs for all circles. (**B**) Distribution of the Log10 adjusted circle length in basepairs for circles with genes. (**C**) Distribution of the Log10 adjusted circle length in basepairs for circles without genes.

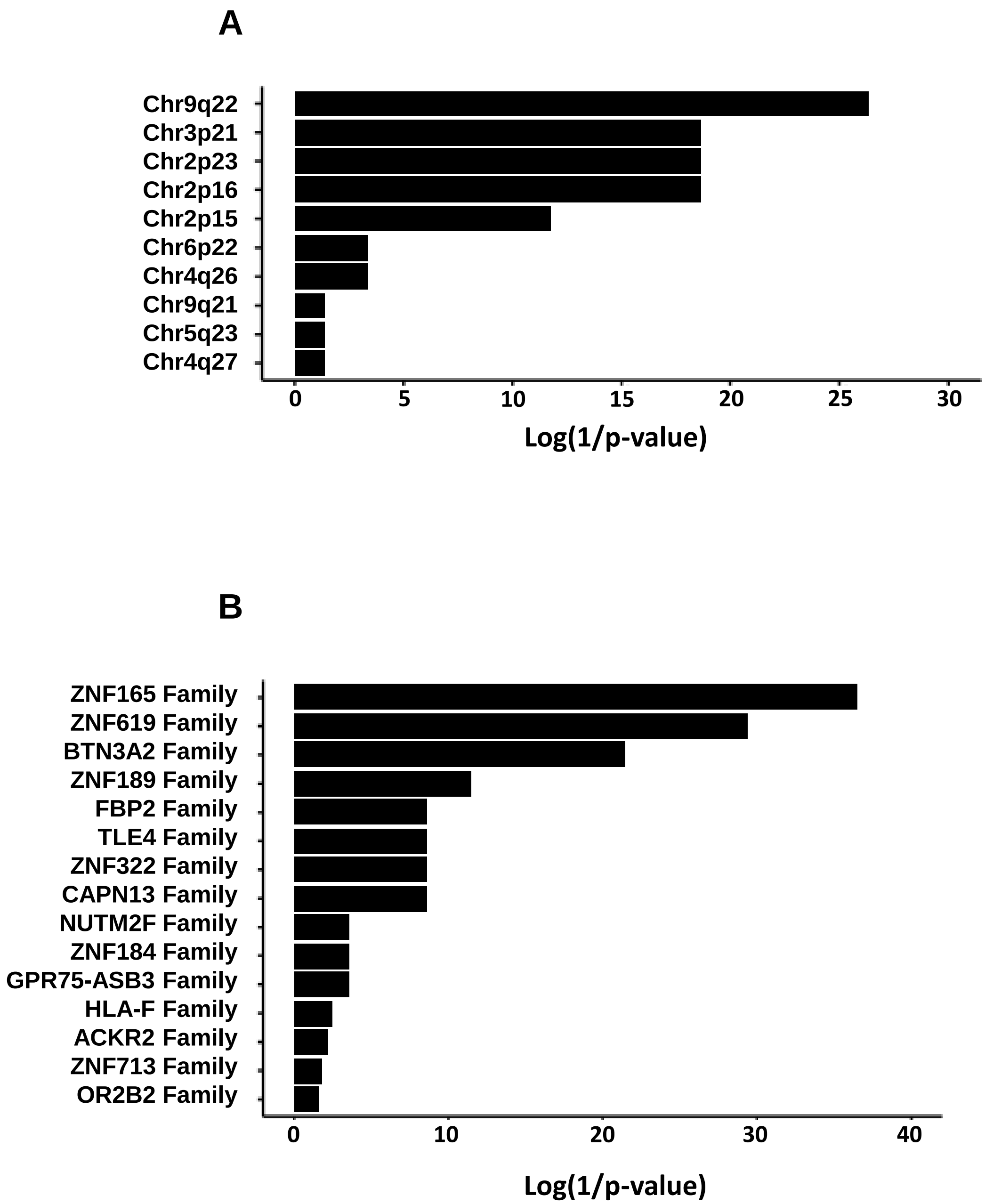

Supplemental Figure S2. Specific chromosomal locations and homologous gene families are enriched for genes commonly found in circles. (**A**) Waterfall plot for the MSigDB chromosome position database, where the length of the bar is the Log10(1/p-value). (**B**) Waterfall plot of homologous-gene families, where the length of the bar is the Log10(1/p-value).

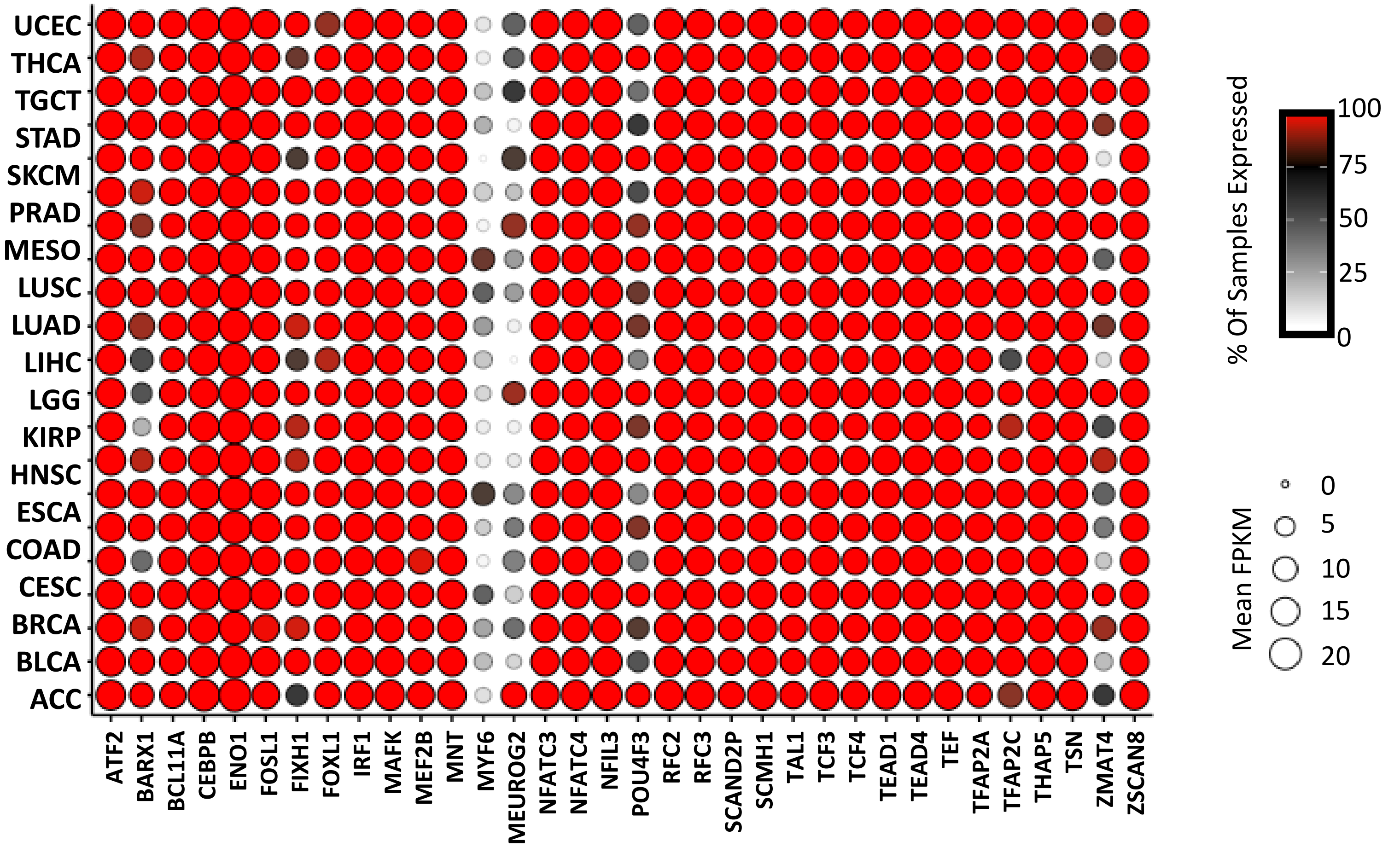

Supplemental Figure S3. Transcription factors with DNA motifs at circle breakpoints are expressed in tumors. (A) Bubble plot of the mean upper quartile normalized FPKM and percent of samples expressing each JASPER transcription factor identified +/-250bp from circle breakpoints. The size of the dots are scaled by the mean FPKM value. The color of the dots are scaled by the percent of tumors that express the transcription factor.

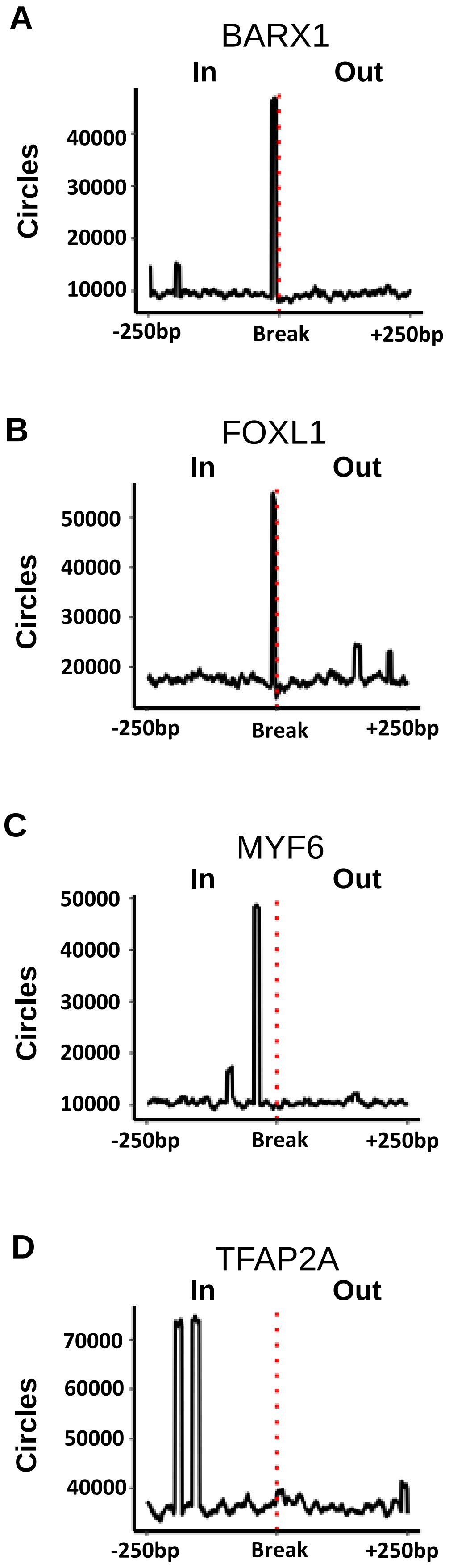

Supplemental Figure S4. Position of motifs enriched inside the circle adjacent to the breakpoint. (**A**) Position of BARX1 with respect to the circle breakpoint. (**B**) Position of FOXL1 with respect to the circle breakpoint. (**C**) Position of MYF6 with respect to the circle breakpoint. (**D**) Position of TFAP2A with respect to the circle breakpoint.

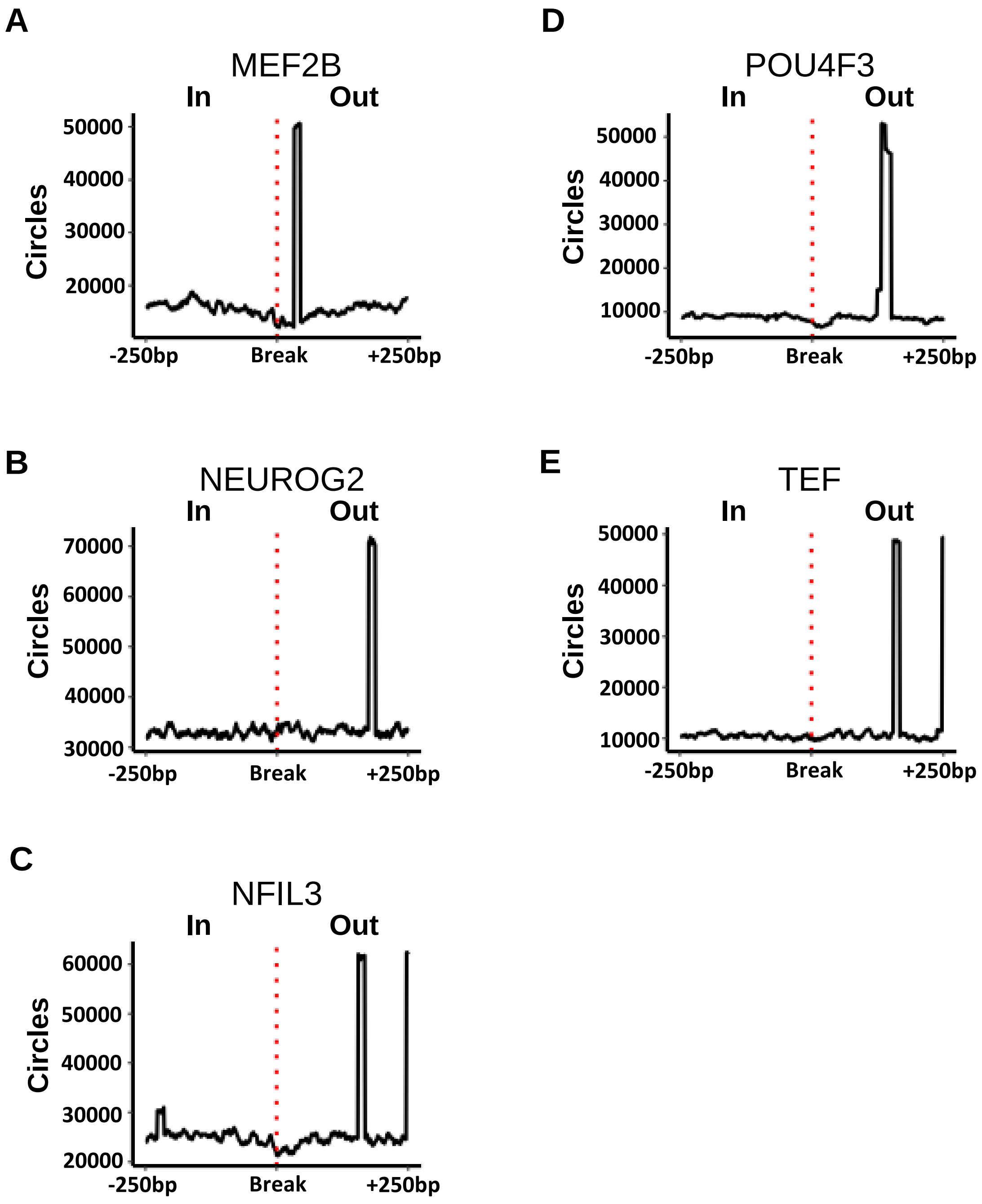

Supplemental Figure S5. Positions of motifs enriched outside the circle adjacent to the breakpoint. (**A**) Position of MEF2B with respect to the circle breakpoint. (**B**) Position of NEUROG2 with respect to the circle breakpoint. (**C**) Position of NFIL3 with respect to the circle breakpoint. (**D**) Position of POU4F3 with respect to the circle breakpoint. (**E**) Position of TEF with respect to the circle breakpoint.

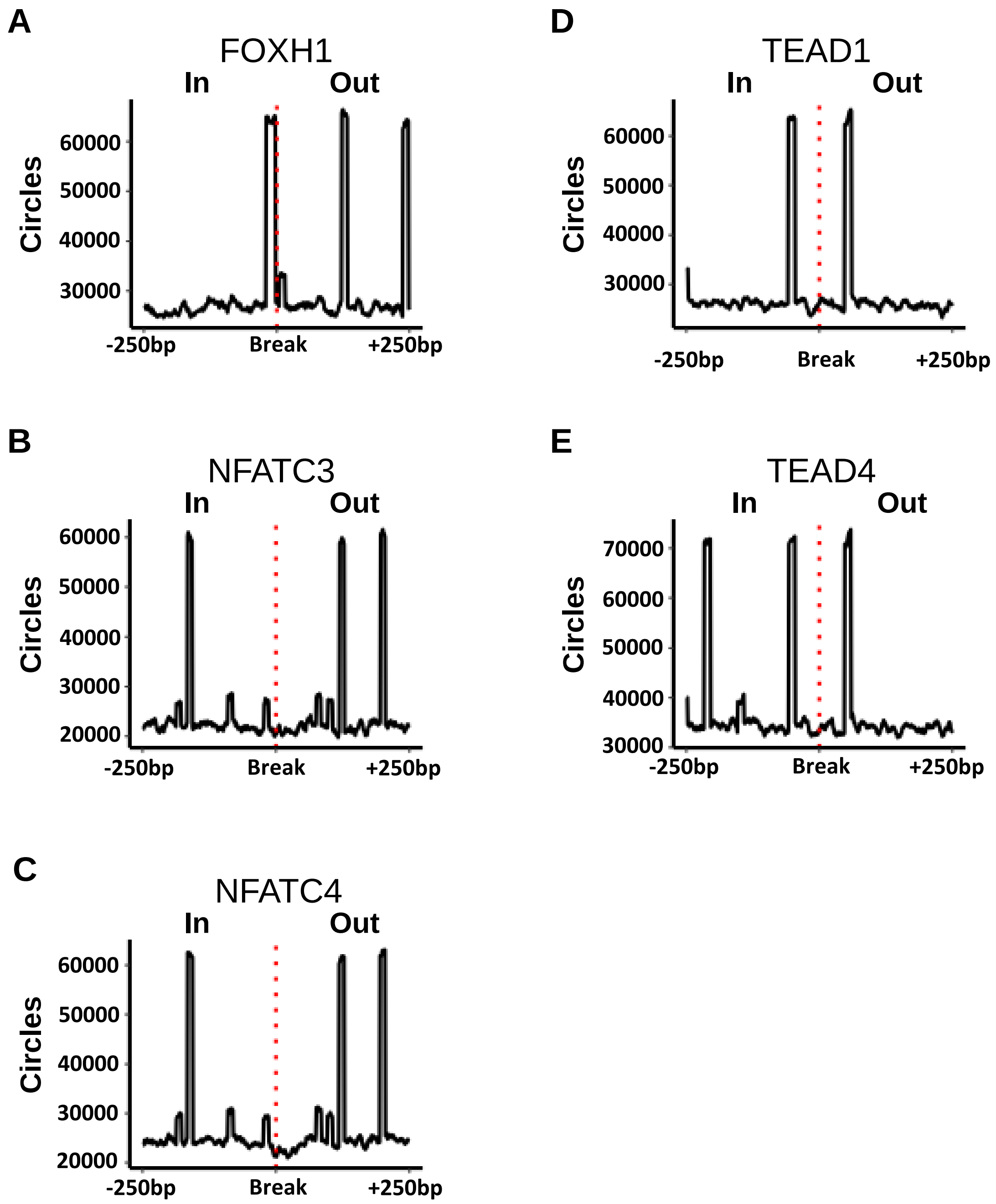

Supplement Figure S6. Positions of motifs enriched on both sides of the breakpoint. (**A**) Position of FOXH1 with respect to the circle breakpoint. (**B**) Position of NFATC3 with respect to the circle breakpoint. (**C**) Position of NFATC4 with respect to the circle breakpoint. (**D**) Position of TEAD1 with respect to the circle breakpoint. (**E**) Position of TEAD4 with respect to the circle breakpoint.

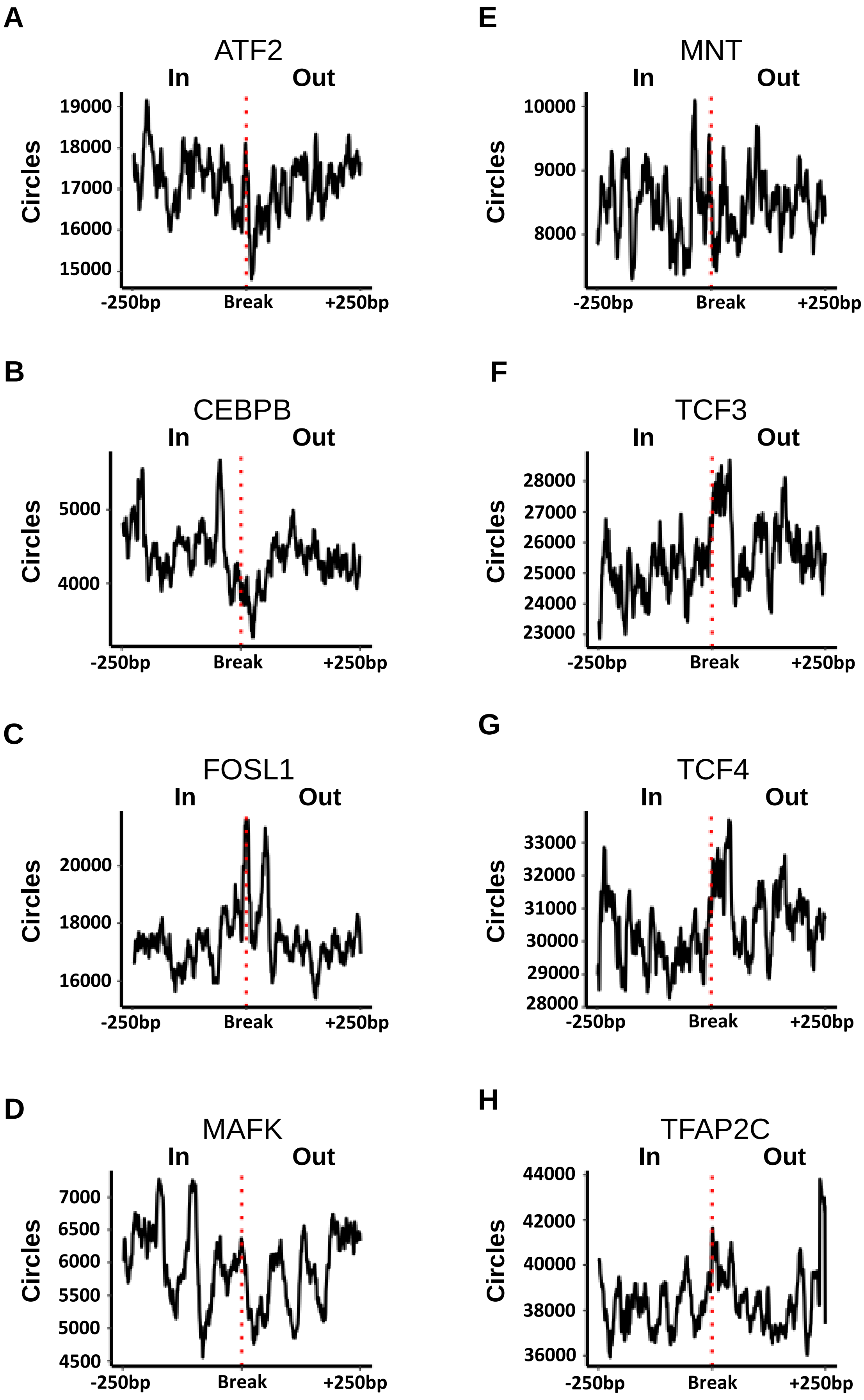

Supplement Figure S7. Positions of motifs enriched surrounding circle ends. (**A**) Position of ATF2 with respect to the circle breakpoint. (**B**) Position of CEBPB with respect to the circle breakpoint. (**C**) Position of FOSL1 with respect to the circle breakpoint. (**D**) Position of MAFK with respect to the circle breakpoint. (**E**) Position of MNT with respect to the circle breakpoint. (**F**) Position of TCF3 with respect to the circle breakpoint. (**G**) Position of TCF4 with respect to the circle breakpoint. (**H**) Position of TFAP2C with respect to the circle breakpoint.
